# *catena*-Poly[[(diiodidocadmium)-μ-{1-[(1*H*-benzimidazol-2-yl)meth­yl]-1*H*-imidazole-κ^2^ *N*:*N*′}] *N*,*N*-dimethyl­formamide monosolvate]

**DOI:** 10.1107/S1600536811050823

**Published:** 2011-11-30

**Authors:** Bingtao Liu, Lei Zhao, Ting Li, Xiangru Meng

**Affiliations:** a School of Environmental and Municipal Engineering, North China Institute of Water Conservancy and Hydroelectric Power, Zhengzhou 450011, People’s Republic of China; b School of Chemical and Engineering, Zhengzhou University, Zhengzhou 450001, People’s Republic of China; c Department of Chemistry, Zhengzhou University, Zhengzhou 450001, People’s Republic of China

## Abstract

In the title complex, {[CdI_2_(C_11_H_10_N_4_)]·C_3_H_7_NO}_*n*_, the Cd^II^ ion is four-coordinated by two N atoms from two 1-[(1*H*-benzimidazol-1-yl)meth­yl]-1*H*-imidazole (bmi) ligands and by two terminal I^−^ anions in a distorted tetra­hedral geometry. One of the two I^−^ anions is disordered over two sets of sites, with refined occupancies of 0.66 (5) and 0.34 (5). The Cd^II^ ions are bridged by bmi ligands, leading to the formation of a chain along [001]. Dimethyl­formamide solvent mol­ecules are located between these chains. Classical N—H⋯O hydrogen bonding between the bmi ligands and the solvent mol­ecules leads to a consolidation of the structure.

## Related literature

For background information on complexes based on *N*-heterocyclic ligands, see: Meng *et al.* (2010 ▶); Mondal *et al.* (2009 ▶); Zhou *et al.* (2011 ▶). 
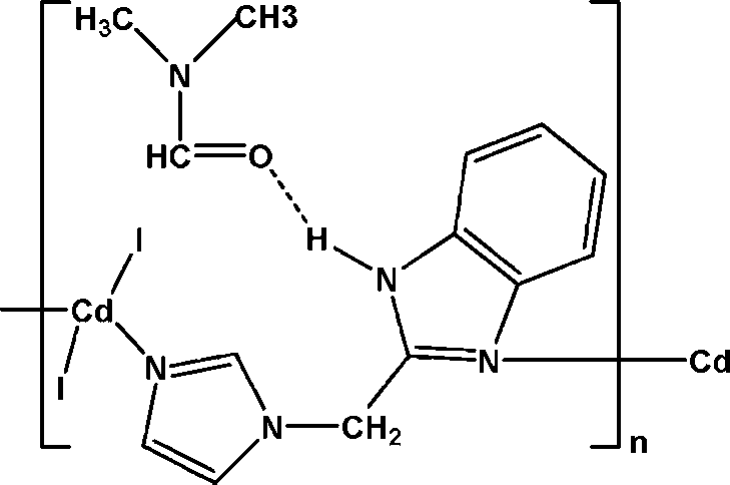


## Experimental

### 

#### Crystal data

[CdI_2_(C_11_H_10_N_4_)]·C_3_H_7_NO
*M* *_r_* = 637.53
Monoclinic, 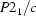

*a* = 7.2216 (14) Å
*b* = 17.181 (3) Å
*c* = 16.374 (3) Å
β = 96.34 (3)°
*V* = 2019.1 (7) Å^3^
*Z* = 4
Mo *K*α radiation
μ = 4.15 mm^−1^
*T* = 293 K
0.15 × 0.12 × 0.10 mm

#### Data collection

Rigaku Saturn diffractometer
Absorption correction: multi-scan (*CrystalClear*; Rigaku/MSC, 2006 ▶) *T*_min_ = 0.575, *T*_max_ = 0.682
16931 measured reflections
3950 independent reflections
3597 reflections with *I* > 2σ(*I*)
*R*_int_ = 0.028

#### Refinement

*R*[*F* ^2^ > 2σ(*F* ^2^)] = 0.035
*wR*(*F* ^2^) = 0.071
*S* = 1.15
3950 reflections
218 parameters
H-atom parameters constrained
Δρ_max_ = 0.42 e Å^−3^
Δρ_min_ = −0.77 e Å^−3^

### 

Data collection: *CrystalClear* (Rigaku/MSC, 2006 ▶); cell refinement: *CrystalClear*; data reduction: *CrystalClear*; program(s) used to solve structure: *SHELXS97* (Sheldrick, 2008 ▶); program(s) used to refine structure: *SHELXL97* (Sheldrick, 2008 ▶); molecular graphics: *XP* in *SHELXTL* (Sheldrick, 2008 ▶); software used to prepare material for publication: *SHELXTL*.

## Supplementary Material

Crystal structure: contains datablock(s) global, I. DOI: 10.1107/S1600536811050823/wm2569sup1.cif

Structure factors: contains datablock(s) I. DOI: 10.1107/S1600536811050823/wm2569Isup2.hkl

Additional supplementary materials:  crystallographic information; 3D view; checkCIF report

## Figures and Tables

**Table 1:** Hydrogen-bond geometry (Å, °)

*D*—H⋯*A*	*D*—H	H⋯*A*	*D*⋯*A*	*D*—H⋯*A*
N3—H3*B*⋯O1	0.86	1.92	2.741 (5)	159

**Fig. 1.**
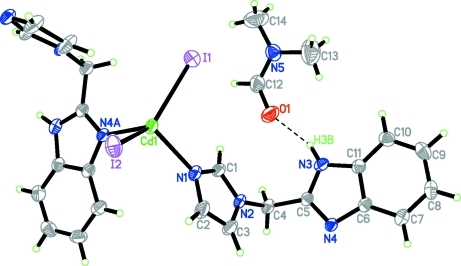
View of the title complex showing atom labelling and 30% probability displacement ellipsolids. Hydrogen bonding is indicated by a dashed line. Only one component of the disordered I1 atom is shown. [Symmetry code A: x, -y + 3/2, z - 1/2.]

**Fig. 2.**
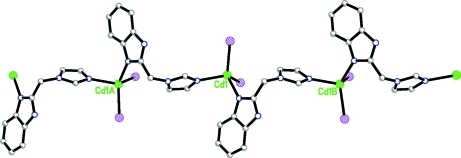
View of the one-dimensional chain in the title complex. [Symmetry codes A: x, -y + 3/2, z - 1/2; B: x, -y + 3/2, z + 1/2.]

## supplementary crystallographic information

### Comment

A large number of complexes based on N-heterocyclic ligands have been synthesized (Meng *et al.*, 2010; Mondal *et al.*, 2009; Zhou *et al.*, 2011). In order to further explore metal-organic frameworks with new structures, we selected 1-[(1*H*-benzimidazole-1-yl)methyl]-1*H*-1,3-imidazole which has abundant N-donor sites to self-assembly with CdI_2_ and obtained the polymeric title complex, {[CdI_2_(C_11_H_10_N_4_)]^.^C_3_H_7_NO}_n_, of which the crystal structure is reported herein.

As shown in Figure 1, the Cd^II^ ion is in a distorted tetrahedral coordination environment defined by two nitrogen atoms from two 1-[(1*H*-benzimidazole-1-yl)methyl]-1*H*-1,3-imidazole (bmi) ligands and two terminal iodine atoms. One of the two iodine atoms is disordered over two positions in a 0.66 (5):0.34 (5) ratio. Each bmi ligand bridges two Cd^II^ ions yielding a chain parallel to [001] with a Cd···Cd distance of 8.2123 (16) Å (Figure 2). In addition, there are N—H···O hydrogen bonds present between benzimidazole groups and *N*,*N*-dimethylformamide solvent molecules.

### Experimental

The ligand 1-[(1*H*-benzimidazole-1-yl)methyl]-1*H*-1,3-imidazole (0.1 mmol) in methanol (4 ml) was added dropwise to an aqueous solution (2 ml) of cadmium iodide (0.1 mmol). The resulting solution was allowed to stand at room temperature. After four weeks colourless crystals of good quality were obtained from the filtrate and dried in air.

### Refinement

The disordered iodine atom was modeled by splitting the atom into two components (I1 and I1'), the site occupation factors of which refined in a ratio of 0.66 (5):0.34 (5). H atoms are positioned geometrically and refined as riding atoms, with C—H = 0.93 (aromatic) Å, 0.96 (—CH_3_) Å, 0.97 (—CH_2_) Å, and N—H = 0.86 Å and with U_iso_(H) = 1.2U_eq_(C,N), 1.5(CH_3_) U_eq_(C).

### Crystal data

**Table:**

[CdI_2_(C_11_H_10_N_4_)]·C_3_H_7_NO	*F*(000) = 1192
*M**_r_* = 637.53	*D*_x_ = 2.097 Mg m^−^^3^
Monoclinic, *P*2_1_/*c*	Mo *K*α radiation, λ = 0.71073 Å
Hall symbol: -P 2ybc	Cell parameters from 5502 reflections
*a* = 7.2216 (14) Å	θ = 2.5–27.8°
*b* = 17.181 (3) Å	µ = 4.15 mm^−^^1^
*c* = 16.374 (3) Å	*T* = 293 K
β = 96.34 (3)°	Prism, colourless
*V* = 2019.1 (7) Å^3^	0.15 × 0.12 × 0.10 mm
*Z* = 4	

### Data collection

**Table:**

Rigaku Saturn diffractometer	3950 independent reflections
Radiation source: fine-focus sealed tube	3597 reflections with *I* > 2σ(*I*)
graphite	*R*_int_ = 0.028
Detector resolution: 28.5714 pixels mm^-1^	θ_max_ = 26.0°, θ_min_ = 2.5°
ω scans	*h* = −8→8
Absorption correction: multi-scan (*CrystalClear*; Rigaku/MSC, 2006)	*k* = −21→21
*T*_min_ = 0.575, *T*_max_ = 0.682	*l* = −20→20
16931 measured reflections	

### Refinement

**Table:**

Refinement on *F*^2^	Primary atom site location: structure-invariant direct methods
Least-squares matrix: full	Secondary atom site location: difference Fourier map
*R*[*F*^2^ > 2σ(*F*^2^)] = 0.035	Hydrogen site location: inferred from neighbouring sites
*wR*(*F*^2^) = 0.071	H-atom parameters constrained
*S* = 1.15	*w* = 1/[σ^2^(*F*_o_^2^) + (0.0274*P*)^2^ + 1.599*P*] where *P* = (*F*_o_^2^ + 2*F*_c_^2^)/3
3950 reflections	(Δ/σ)_max_ = 0.004
218 parameters	Δρ_max_ = 0.42 e Å^−^^3^
0 restraints	Δρ_min_ = −0.77 e Å^−^^3^

### Special details

**Table:**

Geometry. All e.s.d.'s (except the e.s.d. in the dihedral angle between two l.s. planes) are estimated using the full covariance matrix. The cell e.s.d.'s are taken into account individually in the estimation of e.s.d.'s in distances, angles and torsion angles; correlations between e.s.d.'s in cell parameters are only used when they are defined by crystal symmetry. An approximate (isotropic) treatment of cell e.s.d.'s is used for estimating e.s.d.'s involving l.s. planes.
Refinement. Refinement of *F*^2^ against ALL reflections. The weighted *R*-factor *wR* and goodness of fit *S* are based on *F*^2^, conventional *R*-factors *R* are based on *F*, with *F* set to zero for negative *F*^2^. The threshold expression of *F*^2^ > σ(*F*^2^) is used only for calculating *R*-factors(gt) *etc*. and is not relevant to the choice of reflections for refinement. *R*-factors based on *F*^2^ are statistically about twice as large as those based on *F*, and *R*- factors based on ALL data will be even larger.

### Fractional atomic coordinates and isotropic or equivalent isotropic displacement parameters (Å^2^)

**Table:**

	*x*	*y*	*z*	*U*_iso_*/*U*_eq_	Occ. (<1)
Cd1	−0.01312 (4)	0.731203 (18)	0.878202 (17)	0.04345 (10)	
I1	0.1380 (7)	0.5878 (3)	0.9078 (3)	0.0636 (8)	0.67 (5)
I1'	0.108 (4)	0.5857 (7)	0.9160 (12)	0.081 (2)	0.33 (5)
I2	−0.37470 (4)	0.76342 (2)	0.90167 (2)	0.06053 (11)	
N1	0.0248 (5)	0.7542 (2)	0.7464 (2)	0.0472 (9)	
N2	0.1550 (5)	0.75706 (19)	0.63150 (19)	0.0420 (8)	
N3	0.3002 (4)	0.6008 (2)	0.54610 (19)	0.0425 (8)	
H3B	0.3661	0.5896	0.5916	0.051*	
N4	0.1442 (4)	0.67192 (18)	0.44848 (17)	0.0364 (7)	
N5	0.5574 (5)	0.5004 (2)	0.7876 (3)	0.0617 (10)	
C1	0.1732 (6)	0.7355 (2)	0.7110 (2)	0.0442 (10)	
H1A	0.2774	0.7105	0.7375	0.053*	
C2	−0.0939 (7)	0.7889 (3)	0.6863 (3)	0.0598 (13)	
H2A	−0.2118	0.8078	0.6933	0.072*	
C3	−0.0154 (7)	0.7916 (3)	0.6155 (3)	0.0610 (13)	
H3A	−0.0668	0.8127	0.5658	0.073*	
C4	0.2911 (6)	0.7427 (2)	0.5727 (2)	0.0451 (10)	
H4A	0.4138	0.7361	0.6026	0.054*	
H4B	0.2956	0.7877	0.5371	0.054*	
C5	0.2437 (5)	0.6721 (2)	0.5212 (2)	0.0367 (8)	
C6	0.1353 (5)	0.5940 (2)	0.4243 (2)	0.0391 (9)	
C7	0.0492 (6)	0.5583 (3)	0.3534 (3)	0.0514 (11)	
H7A	−0.0167	0.5873	0.3119	0.062*	
C8	0.0650 (6)	0.4791 (3)	0.3471 (3)	0.0574 (12)	
H8A	0.0091	0.4541	0.3004	0.069*	
C9	0.1627 (6)	0.4351 (3)	0.4090 (3)	0.0568 (12)	
H9A	0.1699	0.3815	0.4024	0.068*	
C10	0.2487 (6)	0.4682 (3)	0.4794 (3)	0.0512 (11)	
H10A	0.3135	0.4385	0.5207	0.061*	
C11	0.2335 (5)	0.5487 (2)	0.4856 (2)	0.0412 (9)	
C12	0.5480 (8)	0.5743 (3)	0.7687 (3)	0.0761 (16)	
H12A	0.5960	0.6092	0.8090	0.091*	
C13	0.4896 (11)	0.4450 (5)	0.7255 (5)	0.124 (3)	
H13A	0.4481	0.4720	0.6755	0.187*	
H13B	0.3875	0.4164	0.7437	0.187*	
H13C	0.5879	0.4096	0.7160	0.187*	
C14	0.6277 (11)	0.4732 (5)	0.8671 (4)	0.125 (3)	
H14A	0.6672	0.5168	0.9015	0.188*	
H14B	0.7317	0.4393	0.8627	0.188*	
H14C	0.5316	0.4453	0.8908	0.188*	
O1	0.4814 (5)	0.6021 (2)	0.7023 (2)	0.0803 (11)	

### Atomic displacement parameters (Å^2^)

**Table:**

	*U*^11^	*U*^22^	*U*^33^	*U*^12^	*U*^13^	*U*^23^
Cd1	0.05384 (19)	0.04766 (19)	0.03022 (16)	−0.00195 (14)	0.01071 (13)	−0.00394 (12)
I1	0.0812 (19)	0.0510 (9)	0.0590 (10)	0.0095 (5)	0.0096 (6)	−0.0014 (9)
I1'	0.126 (5)	0.0482 (13)	0.067 (2)	0.011 (3)	0.006 (3)	0.0078 (14)
I2	0.05041 (19)	0.0619 (2)	0.0716 (2)	−0.00441 (14)	0.01688 (15)	−0.01633 (16)
N1	0.059 (2)	0.054 (2)	0.0300 (17)	0.0094 (17)	0.0098 (16)	−0.0026 (15)
N2	0.052 (2)	0.049 (2)	0.0251 (16)	0.0037 (16)	0.0056 (14)	−0.0033 (14)
N3	0.0425 (18)	0.054 (2)	0.0317 (17)	0.0087 (16)	0.0062 (14)	0.0063 (15)
N4	0.0402 (17)	0.0411 (19)	0.0287 (16)	0.0022 (14)	0.0070 (13)	−0.0006 (14)
N5	0.060 (2)	0.056 (3)	0.067 (3)	0.000 (2)	−0.004 (2)	−0.009 (2)
C1	0.049 (2)	0.052 (2)	0.032 (2)	0.011 (2)	0.0064 (17)	0.0023 (18)
C2	0.056 (3)	0.085 (4)	0.039 (2)	0.023 (3)	0.004 (2)	−0.009 (2)
C3	0.070 (3)	0.083 (4)	0.029 (2)	0.031 (3)	0.002 (2)	0.001 (2)
C4	0.049 (2)	0.054 (3)	0.033 (2)	−0.0058 (19)	0.0099 (18)	−0.0033 (18)
C5	0.0346 (19)	0.046 (2)	0.0309 (19)	0.0018 (17)	0.0112 (15)	−0.0017 (17)
C6	0.036 (2)	0.049 (2)	0.033 (2)	0.0018 (17)	0.0114 (16)	−0.0012 (17)
C7	0.054 (3)	0.061 (3)	0.039 (2)	−0.002 (2)	0.0052 (19)	−0.005 (2)
C8	0.064 (3)	0.056 (3)	0.053 (3)	−0.011 (2)	0.013 (2)	−0.019 (2)
C9	0.059 (3)	0.043 (3)	0.073 (3)	−0.001 (2)	0.026 (3)	−0.006 (2)
C10	0.054 (3)	0.048 (3)	0.054 (3)	0.008 (2)	0.015 (2)	0.004 (2)
C11	0.041 (2)	0.047 (2)	0.038 (2)	0.0049 (18)	0.0134 (17)	0.0013 (18)
C12	0.091 (4)	0.070 (4)	0.062 (3)	0.016 (3)	−0.015 (3)	−0.014 (3)
C13	0.139 (7)	0.114 (6)	0.119 (6)	−0.035 (5)	0.007 (5)	−0.035 (5)
C14	0.149 (7)	0.121 (7)	0.095 (5)	0.001 (5)	−0.034 (5)	0.037 (5)
O1	0.092 (3)	0.100 (3)	0.048 (2)	0.036 (2)	−0.0028 (18)	0.0057 (19)

### Geometric parameters (Å, °)

**Table:**

Cd1—N1	2.241 (3)	C3—H3A	0.9300
Cd1—N4^i^	2.258 (3)	C4—C5	1.496 (5)
Cd1—I1'	2.698 (12)	C4—H4A	0.9700
Cd1—I1	2.716 (6)	C4—H4B	0.9700
Cd1—I2	2.7375 (7)	C6—C7	1.396 (6)
N1—C1	1.313 (5)	C6—C11	1.400 (5)
N1—C2	1.369 (6)	C7—C8	1.372 (6)
N2—C1	1.346 (5)	C7—H7A	0.9300
N2—C3	1.365 (5)	C8—C9	1.392 (7)
N2—C4	1.471 (5)	C8—H8A	0.9300
N3—C5	1.339 (5)	C9—C10	1.371 (6)
N3—C11	1.383 (5)	C9—H9A	0.9300
N3—H3B	0.8600	C10—C11	1.392 (6)
N4—C5	1.321 (5)	C10—H10A	0.9300
N4—C6	1.395 (5)	C12—O1	1.235 (6)
N4—Cd1^ii^	2.258 (3)	C12—H12A	0.9300
N5—C12	1.307 (7)	C13—H13A	0.9600
N5—C14	1.424 (7)	C13—H13B	0.9600
N5—C13	1.438 (7)	C13—H13C	0.9600
C1—H1A	0.9300	C14—H14A	0.9600
C2—C3	1.346 (6)	C14—H14B	0.9600
C2—H2A	0.9300	C14—H14C	0.9600
			
N1—Cd1—N4^i^	104.65 (12)	C5—C4—H4B	109.2
N1—Cd1—I1'	108.3 (6)	H4A—C4—H4B	107.9
N4^i^—Cd1—I1'	115.7 (3)	N4—C5—N3	112.9 (3)
N1—Cd1—I1	104.02 (15)	N4—C5—C4	125.3 (4)
N4^i^—Cd1—I1	114.07 (14)	N3—C5—C4	121.8 (3)
I1'—Cd1—I1	5.7 (7)	N4—C6—C7	131.3 (4)
N1—Cd1—I2	108.64 (10)	N4—C6—C11	109.0 (3)
N4^i^—Cd1—I2	102.34 (8)	C7—C6—C11	119.7 (4)
I1'—Cd1—I2	116.4 (7)	C8—C7—C6	117.7 (4)
I1—Cd1—I2	121.94 (12)	C8—C7—H7A	121.1
C1—N1—C2	105.5 (3)	C6—C7—H7A	121.1
C1—N1—Cd1	125.0 (3)	C7—C8—C9	121.6 (4)
C2—N1—Cd1	129.5 (3)	C7—C8—H8A	119.2
C1—N2—C3	107.2 (3)	C9—C8—H8A	119.2
C1—N2—C4	125.9 (4)	C10—C9—C8	122.3 (4)
C3—N2—C4	126.9 (3)	C10—C9—H9A	118.9
C5—N3—C11	107.7 (3)	C8—C9—H9A	118.9
C5—N3—H3B	126.2	C9—C10—C11	116.1 (4)
C11—N3—H3B	126.2	C9—C10—H10A	121.9
C5—N4—C6	105.2 (3)	C11—C10—H10A	121.9
C5—N4—Cd1^ii^	130.6 (3)	N3—C11—C10	132.2 (4)
C6—N4—Cd1^ii^	123.9 (2)	N3—C11—C6	105.2 (3)
C12—N5—C14	122.6 (5)	C10—C11—C6	122.6 (4)
C12—N5—C13	118.1 (5)	O1—C12—N5	126.1 (5)
C14—N5—C13	119.2 (6)	O1—C12—H12A	116.9
N1—C1—N2	111.2 (4)	N5—C12—H12A	116.9
N1—C1—H1A	124.4	N5—C13—H13A	109.5
N2—C1—H1A	124.4	N5—C13—H13B	109.5
C3—C2—N1	110.1 (4)	H13A—C13—H13B	109.5
C3—C2—H2A	124.9	N5—C13—H13C	109.5
N1—C2—H2A	124.9	H13A—C13—H13C	109.5
C2—C3—N2	106.0 (4)	H13B—C13—H13C	109.5
C2—C3—H3A	127.0	N5—C14—H14A	109.5
N2—C3—H3A	127.0	N5—C14—H14B	109.5
N2—C4—C5	112.1 (3)	H14A—C14—H14B	109.5
N2—C4—H4A	109.2	N5—C14—H14C	109.5
C5—C4—H4A	109.2	H14A—C14—H14C	109.5
N2—C4—H4B	109.2	H14B—C14—H14C	109.5
			
N4^i^—Cd1—N1—C1	80.6 (4)	C11—N3—C5—N4	−0.1 (4)
I1'—Cd1—N1—C1	−43.3 (7)	C11—N3—C5—C4	179.7 (3)
I1—Cd1—N1—C1	−39.4 (4)	N2—C4—C5—N4	92.8 (4)
I2—Cd1—N1—C1	−170.6 (3)	N2—C4—C5—N3	−87.0 (4)
N4^i^—Cd1—N1—C2	−99.6 (4)	C5—N4—C6—C7	179.9 (4)
I1'—Cd1—N1—C2	136.4 (7)	Cd1^ii^—N4—C6—C7	6.0 (6)
I1—Cd1—N1—C2	140.4 (4)	C5—N4—C6—C11	0.0 (4)
I2—Cd1—N1—C2	9.1 (4)	Cd1^ii^—N4—C6—C11	−173.9 (2)
C2—N1—C1—N2	0.4 (5)	N4—C6—C7—C8	−179.9 (4)
Cd1—N1—C1—N2	−179.8 (3)	C11—C6—C7—C8	0.0 (6)
C3—N2—C1—N1	0.0 (5)	C6—C7—C8—C9	0.2 (6)
C4—N2—C1—N1	−177.6 (4)	C7—C8—C9—C10	−0.1 (7)
C1—N1—C2—C3	−0.7 (6)	C8—C9—C10—C11	−0.2 (6)
Cd1—N1—C2—C3	179.5 (3)	C5—N3—C11—C10	−179.4 (4)
N1—C2—C3—N2	0.7 (6)	C5—N3—C11—C6	0.0 (4)
C1—N2—C3—C2	−0.4 (6)	C9—C10—C11—N3	179.9 (4)
C4—N2—C3—C2	177.2 (4)	C9—C10—C11—C6	0.5 (6)
C1—N2—C4—C5	97.1 (5)	N4—C6—C11—N3	0.0 (4)
C3—N2—C4—C5	−80.1 (6)	C7—C6—C11—N3	−180.0 (3)
C6—N4—C5—N3	0.1 (4)	N4—C6—C11—C10	179.5 (3)
Cd1^ii^—N4—C5—N3	173.4 (2)	C7—C6—C11—C10	−0.4 (6)
C6—N4—C5—C4	−179.7 (3)	C14—N5—C12—O1	−176.6 (6)
Cd1^ii^—N4—C5—C4	−6.4 (5)	C13—N5—C12—O1	1.9 (9)

Symmetry codes: (i) *x*, −*y*+3/2, *z*+1/2; (ii) *x*, −*y*+3/2, *z*−1/2.

### Hydrogen-bond geometry (Å, °)

**Table:**

*D*—H···*A*	*D*—H	H···*A*	*D*···*A*	*D*—H···*A*
N3—H3B···O1	0.86	1.92	2.741 (5)	159.
